# Meta-analysis of associations between childhood adversity and hippocampus and amygdala volume in non-clinical and general population samples

**DOI:** 10.1016/j.nicl.2017.02.016

**Published:** 2017-02-22

**Authors:** Maria Calem, Konstantinos Bromis, Philip McGuire, Craig Morgan, Matthew J Kempton

**Affiliations:** a Department of Psychosis Studies, Institute of Psychiatry, Psychology and Neuroscience, King's College London, London SE5 8AF, UK; b School of Electrical and Computer Engineering, National Technical University of Athens, Greece; c Health Services and Population Research Department, Institute of Psychiatry, Psychology and Neuroscience, King's College London, London SE5 8AF, UK; d Department of Neuroimaging, Institute of Psychiatry, Psychology and Neuroscience, King's College London, London SE5 8AF, UK

**Keywords:** Stress, Neuroimaging, Adversity, Child abuse

## Abstract

**Background:**

Studies of psychiatric populations have reported associations between childhood adversity and volumes of stress-related brain structures. This meta-analysis investigated these associations in non-clinical samples and therefore independent of the effects of severe mental health difficulties and their treatment.

**Methods:**

The MEDLINE database was searched for magnetic resonance imaging studies measuring brain structure in adults with and without childhood adversity. Fifteen eligible papers (1781 participants) reporting hippocampal volumes and/or amygdala volumes were pooled using a random effects meta-analysis.

**Results:**

Those with childhood adversity had lower hippocampus volumes (hedges g = − 0.15, *p* = 0.010). Controlling for gender, this difference became less evident (hedges g = − 0.12, *p* = 0.124). This association differed depending on whether studies included participants with some psychopathology, though this may be due to differences in the type of adversity these studies examined. There was no strong evidence of any differences in amygdala volume.

**Discussion:**

Childhood adversity may have only a modest impact on stress-related brain structures in those without significant mental health difficulties.

## Highlights

• Reduced hippocampal volume found in non-clinical people with childhood adversity
• This effect was less marked than those found in psychiatric populations.
• There was some evidence for confounding by gender.
• Further studies needed on impact of different forms of adversity on neuroanatomy.

## 1 Introduction

Childhood adversity, defined as difficult and unpleasant situations and experiences in childhood including physical, sexual, or emotional abuse, neglect and poverty, is highly prevalent worldwide (Kessler et al., 2010). In a recent UK survey (Radford et al., 2013), 24.5% of young adults reported experiencing abuse or neglect by a parent or caregiver during childhood. Childhood abuse and neglect is associated with a range of negative physical and mental health outcomes (see (Wilson, 2010) and (Norman et al., 2012) for reviews), including posttraumatic stress disorder (Kessler et al., 1995), psychosis (Varese et al., 2012), depression and anxiety (Lindert et al., 2014), diabetes (Huffhines et al., 2016) and obesity (Danese and Tan, 2014). Growing up in poverty, another highly prevalent form of childhood adversity, is also related to a range of negative health consequences (Schickedanz et al., 2015).

These negative health outcomes may be due in part to the effects of childhood adversity on brain development. Childhood adversity can be a form of stress at a time where brains are especially sensitive to the neurotoxic effects of excessive release of stress hormones and stress-related epigenetic changes (Lupien et al., 2009). Evidence for altered neurodevelopment comes from several studies that have now shown associations between childhood adversity and neuroanatomical changes (for reviews, see (Hart and Rubia, 2012) and (McLaughlin et al., 2014)). The neurotoxic effect of stress has been demonstrated experimentally in animal studies and importantly, the neuroanatomical effects of stress in these studies are similar to those found to be related to childhood adversity in humans, particularly in the hippocampus and corpus callosum (Teicher et al., 2006).

Research on the neuroanatomy of childhood adversity has often been carried out in samples recruited for their mental health difficulties, for example, demonstrating altered volumes in stress-related brain structures such as the hippocampus and the amygdala in people with post-traumatic stress disorder (Bremner et al., 1997), depression (Vythilingam et al., 2002) and psychosis (Hoy et al., 2012); (Aas et al., 2012). It must be noted that the majority of people who experience childhood adversity do not go on to develop psychiatric illness, though they are of course at much higher risk of doing so (Macmillan et al., 2001). The present study focuses on general population samples, and control groups without a psychiatric disorder included in case-control studies. This is in order to examine whether evidence of the impact of childhood adversity on neuroanatomy can be detected in the absence of selection for mental health difficulties. Childhood adversity may have less impact on brain structures of those who do not go on to develop mental health difficulties, i.e. those resilient to the development of mental ill health in the face of childhood adversity may also have been less affected on a neuroanatomical level. If so, a meta-analytical approach is ideal as it provides increased statistical power to detect more subtle effects. This is particularly relevant for control groups of case-control studies as these individuals have relatively low levels of childhood adversity (Chaney et al., 2014). The focus on non-psychiatric samples also allows for the effect of childhood adversity to be investigated unconfounded by the stress of experiencing severe mental health difficulties and the effect of receiving treatment for these difficulties (such as psychotropic medication and hospitalisation). Overall, this study will therefore allow us to examine whether neuroanatomical changes associated with childhood adversity and psychopathology are not simply a consequence of experiencing or receiving treatment for mental health difficulties.

There are important differences in prevalence of childhood adversity by gender, particularly childhood sexual abuse, which is more common in females (Barth et al., 2013). In addition, women on average have lower hippocampal (Tan et al., 2016) and amygdala (Goldstein et al., 2001) volumes. Therefore, gender could confound the relationship between brain volumes and childhood adversity.

The current study aimed to clarify the impact of childhood adversity on brain structure in a large number of diverse non-psychiatric samples and to present the results adjusted for gender. This was accomplished by conducting a meta-analysis to estimate the association between childhood adversity and volume of specific brain structures in general population or control samples. Specific regions of interest were included if they were reported in enough studies and were highlighted as potentially relevant in preclinical literature. Gender was taken into account as a potential confounder.

## 2 Methods

### 2.1 Study database

Included in the study database were peer-reviewed studies that measured the volume of specific regions of interest using Magnetic Resonance Imaging (MRI) in control or general population samples of working age adults with and without a history of childhood adversity. Medline was searched up to 24th April 2015 using a combination of relevant expanded subject headings and free text searches (see supplementary material for detailed search terms). In total, 1458 records of publications were initially examined. Three studies were later identified via the references of included papers. Studies were excluded if they were case studies or reviews, if they concerned traumatic head injury rather than adversity, if they did not include volumetric data as means and standard deviations (for example, voxel-based morphometry studies that only reported co-ordinates), if they were not studies of working age adults, or if the sample overlapped with another larger study sample. The amygdala and hippocampus were the most commonly studied regions and have previously been highlighted by animal studies as regions effected by psychosocial stress and were therefore chosen for analysis. Fifteen publications fulfilled the inclusion criteria and were included in the database (see PRISMA Flowdiagram in Fig. 1 in supplementary material). Authors were contacted for more information if their paper indicated that they had collected but not reported the relevant data.

**Fig. 1:**
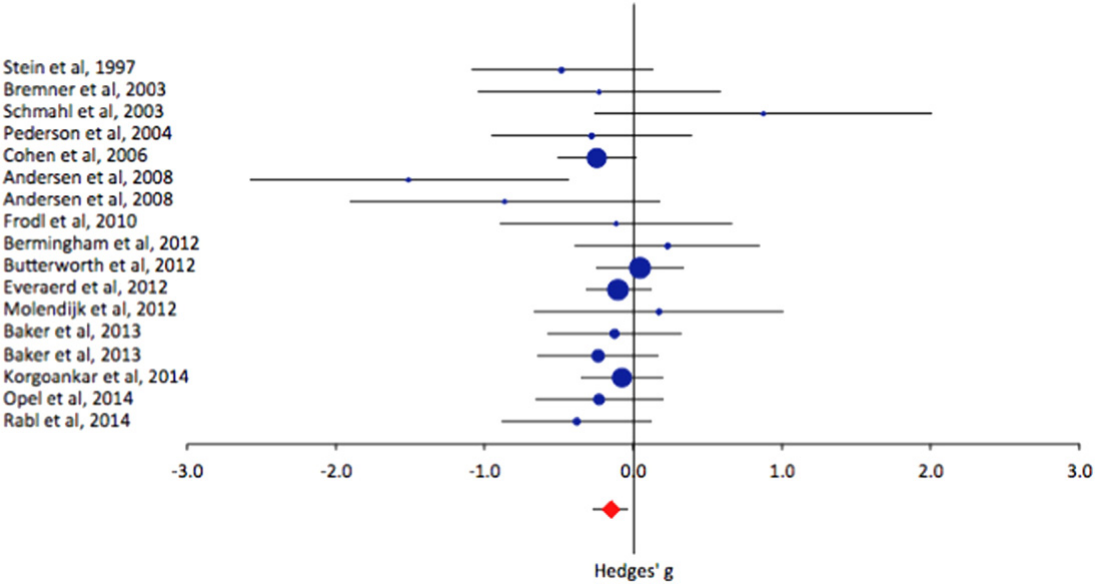
Forest plots showing effect sizes of studies of hippocampal volume differences between healthy control with and without a history of childhood adversity, not controlling gender. Positive effect sizes indicate the region has increased volume in those with childhood adversity, negative effects sizes indicate the region has decreased volume in those with childhood adversity. For each study, the circle indicates the effect size, and the horizontal lines indicate the 95% confidence intervals. The size of the circle represents the relative weight of the particular study in the overall meta-analysis. The diamond at the bottom of each graph represents the overall effect calculated using a random effects model.

The following data were recorded from each study where available: number of subjects with and without a history of childhood adversity, type of childhood adversity studied, mean age of participants at interview and at time of adversity, percentage of female participants, percentage of participants with a psychiatric illness, severity of adversity experienced and mean and standard deviation for hippocampus and amygdala volume.

### 2.2 Defining childhood adversity

Childhood adversity was defined as any difficult and unpleasant situations and experiences in childhood. Studies captured this using either specific measures of trauma, abuse, neglect, poverty or more general measures of adversity and early life stress. Abuse can be physical, sexual or emotional and is usually defined as the action of intentionally harming another person. Neglect is usually defined as failure to provide for a child's basic physical or emotional needs. Studies differed in what form of adversity they focussed on and in how they defined each form of adversity. These details were recorded to check that each studied captured at least one significant form of childhood adversity according to the above definitions. Three studies focussed on sexual abuse, six on multiple forms of abuse, three were of stressful events in early life, two were of abuse and neglect and one study was of poverty. Different cut-offs for childhood were used on the studies, ranging from 12 to 18 years of age.

### 2.3 Hippocampal and amygdala volume meta-analysis

Of the 15 studies in the database, all 15 reported necessary data for hippocampal volume analysis (number of controls with and without childhood adversity, mean and SD volume) and 7 reported the necessary data for the amygdala volume analysis.

### 2.4 Combining study estimates

Hedges g was used, which is Cohen's effect size with a correction for bias from small sample sizes (Hedges and Olkin, 1985). The percentage difference effect size is also specified to aid interpretation of the data (McDonald et al., 2004). A meta-analysis for each brain structure was performed in Excel for Mac 2011 using identical meta-analytical equations used by the METAN command in STATA 9.2 (StatCorp 2006) (Kempton et al., 2011). Outcome measures were combined using a random effects inverse weighted variance model (DerSimonian and Laird, 1986). To minimise the number of comparisons, the analysis was based on the whole (bilateral) volume for each brain structure. Where the left and right volumes were reported in a paper but not the total volume, we ascertained measurements of the total volume using a method (Koolschijn et al., 2009) that requires an estimate of the correlation coefficient between the left and right volumes. This was set as 0.8 but was varied in the sensitivity analysis (see Sensitivity analysis section below). Two studies reported measurements from two subgroups: an early childhood adversity and a late childhood adversity group. We included these subgroups as separate studies. To prevent double counting of the control group in these two instances the number of people in the non-adversity group was calculated as the sample size of the non-adversity group divided by two.

### 2.5 Accounting for gender differences

As women are more likely to experience sexual abuse in childhood than men (Pereda et al., 2009), overall women may have higher rates of childhood adversity. Women also tend to have smaller volume of brain structures including the hippocampus (Tan et al., 2016). This could lead to the spurious finding of childhood adversity being associated with smaller brain volumes because of the confounding effect of gender. Therefore, it was necessary to control for gender as a confounder in the analysis. This was achieved by, within the same analysis, comparing women with adversity to women without adversity, and men with adversity to men without adversity. Thus for studies with both women and men, two effect sizes were calculated and were included in the meta-analysis. Of the 15 papers included, authors provided data separately by gender for 11 studies. To make this analysis by gender more comparable to the main analysis, the main analysis was rerun with just these 11 studies for whom data by gender was available.

### 2.6 Accounting for psychopathology

Studies were divided into three groups depending on whether they excluded their control or general population participants based on psychiatric history. Group A) No psychiatric disorders in adversity or non-adversity group: nine studies excluded participants if they met criteria for a psychiatric disorder. Group B) Psychiatric disorders present in adversity and non-adversity group: three studies did not exclude participants on the basis of psychiatric history. Group C) Psychiatric disorders present in adversity group but not in non-adversity group: in three studies, participants with a history of adversity were included regardless of psychiatric history, while their non-adversity counterparts were included only if they had no such history. As above, to control for gender, a further meta-analysis was run with effect sizes for each study calculated by gender.

### 2.7 Assessing between-study heterogeneity

To test for between-study heterogeneity, Cochran's Q test statistic was calculated (Sutton and Abrams, 2001). The I^2^ statistic, which is equal to the percentage of total variation across studies due to heterogeneity, was also calculated to aid interpretability of between-study heterogeneity (Higgins et al., 2003).

### 2.8 Small study bias

The effect of small study bias (which may include publication bias) was investigated for regions where the pooled effect size revealed a significant difference between groups. Small study bias was assessed using Egger's regression test (Egger et al., 1997).

### 2.9 Sensitivity analysis of methodological issues

To test how robust the results were to variations in the meta-analysis methodology, the effect of the following factors were examined: a) percentage difference in group mean volumes as an outcome measure for continuous data (the calculation of this effect size and the effect size variance has been described in more detail in previous meta-analytical studies);(McDonald et al., 2004, Wright et al., 2000) and b) setting the correlation coefficient between the left and right regional volumes as 0.1, 0.5 and 1.

## 3 Results

### 3.1 Characteristics of included studies

The fifteen studies included in the meta-analysis are listed in Table 1 (for further information on the studies see Supplementary Table 1). The studies included a total of 783 people with a history of childhood adversity and 998 people without. Ten studies used a 1.5 Tesla scanner and five used a 3 Tesla scanner with a mean MRI scan slice thickness of 1.50 mm (SD = 1.0 mm). The cut-off age for defining childhood was 12 and 14 in one study each, 16 and 18 in three studies each and 17 in six studies (not reported by 1 study). Mean age at interview was 33.5 years. The mean proportion of women was 71.6% (reported by 14 studies) with a similar proportion of women in the adversity group (mean = 72.4%) and no adversity group (mean = 72.8%) (reported separately by 12 studies). Hippocampal and/or amygdala volumes were obtained separately by gender from eleven of the fifteen studies. This subsample of 11 studies included 505 people with a history of childhood adversity (336 women, 169 men) and 929 people without (567 women, 362 men).

**Table 1:** List of studies included in the meta-analysis.

	Study	n with CA	n without CA	Brain region (hc/am)	Definition of CA	CA measure	Included in analysis by gender?	Psycho-pathology group^a^	Psychiatric exclusion criteria
1	Stein et al., 1997	21	21	hc	Attempted or completed vaginal or anal penetration occurring between a child 14 years of age or younger and a perpetrator who was at least 5 years older than the child.	Telephone interview	Yes	C	Non-victimized controls were required to be free of current Axis I pathology.
2	Bremner et al., 2003	12	11	hc	Childhood sexual abuse (rape, attempted rape or molestation) before the age of 18	Early trauma inventory	Yes	C	Any serious medical or neurological illness, organic mental disorders, comorbid psychotic disorder. Those with no adversity had no past or present psychiatric diagnosis.
3	Schmahl et al., 2003	5	10	hc, am	Childhood physical or sexual abuse before the age of 18	Early trauma inventory	Yes	B	Any current Axis 1 diagnosis.
4	Pederson et al., 2004	17	17	hc	Severe to extreme childhood physical, emotional or sexual abuse before the age of 17	Childhood trauma questionnaire	Yes	B	Clinically significant alcohol dependence, drug dependence, bipolar, delusional, and thought disorder subscale scores of the MCMI-III.
5	Cohen et al., 2006	143	84	hc, am	Two or more stressful and/or traumatic adverse events before age of 12	Early life stress questionnaire	No	A	Any past or present psychiatric diagnosis
6	Andersen et al., 2008	21	14	hc	Three or more episodes of forced contact childhood sexual abuse, defined as forced involuntary contact with sexual body parts accompanied by either threats of harm to self or others or feelings of fear or terror	Traumatic antecedents questionnaire	Yes	C	No psychiatric exclusion criteria except past or present alcohol/substance. Those with no adversity had no past or present psychiatric diagnosis.
7	Frodl et al., 2010	10	17	hc	High childhood abuse or neglect based on a median split	Childhood trauma questionnaire	No	A	Any past or present psychiatric diagnosis
8	Bermingham et al., 2012	15	29	hc	Presence of childhood abuse or neglect based on CTQ cut-offs	Childhood trauma questionnaire	No	A	Any past or present psychiatric diagnosis
9	Butterworth et al., 2012	50	353	hc, am	Growing up in poverty	Hardship items from the personality and total health questionnaire	Yes	B	None.
10	Everaerd et al., 2012	247	110	hc	At least one life event before the age of 16 likely to have been relatively frequent and entailed relatively high long-term threat	List of threatening life events (Brugha et al., 1985)	Yes	A	Any past or present psychiatric diagnosis
11	Molendijk et al., 2012	7	24	hc	Psychological, physical or sexual abuse or emotional neglect by age of 16	Semi-structured childhood trauma interview	Yes	A	Any past or present (Axis-1) psychiatric diagnosis
12	Baker et al., 2013	97	76	hc, am	Physical, emotional or sexual abuse or other traumatic experiences by age of 18	Early life stress questionnaire	No	A	Any past or present psychiatric diagnosis
13	Korgaonkar et al., 2013	74	150	hc, am	3 or more life stressors known to have a psychological, including abuse, neglect, family conflict, illness/death and natural disasters by age of 18	Early life stress questionnaire	Yes	A	Any past or present psychiatric diagnosis
14	Opel et al., 2014	22	36	hc, am	Sexual, physical or emotional abuse or emotional or physical neglect by age of 17	Childhood trauma questionnaire	Yes	A	Any past or present psychiatric diagnosis
15	Rabl et al., 2014	30	31	hc, am	Sexual, physical or emotional abuse or emotional or physical neglect by age of 17	Childhood trauma questionnaire	Yes	A	Any past or present psychiatric diagnosis
	TOTAL	783	998						

Abbreviations: CA + childhood adversity positive. CA − childhood adversity negative. hc hippocampus. am amygdala. DSM-IV Diagnostic and Statistical Manual of Mental Disorders, 4th edition.

a Studies were grouped by whether they allowed psychopathology in participants with and without childhood adversity: Group A = N/N; Group B = Y/Y, Group C = Y/N.

Exclusion criteria relating to psychiatric history are also described in Table 1. As mentioned above, nine studies (Group A) excluded participants with a current or past psychiatric diagnosis. Three studies (Group B) allowed for some psychiatric history in all participants; of these, one study did not exclude on the basis of psychiatric history at all, one only allowed for past (but not current) Axis 1 diagnoses, and one excluded for more significant substance use or psychiatric symptoms (bipolar, delusional and thought disorder). The three remaining studies (Group C) allowed for some psychiatric history in the adversity group but not in the non-adversity group; one excluded non-adversity group participants with current Axis 1 pathology, and the other two excluded those with any past or present psychiatric diagnosis.

The three groups by psychopathology differed on what type of adversity they focussed on. Of the nine studies in Group A (no psychopathology), four were of multiple forms of abuse, three were of early life stress and two were of abuse or neglect. Of the three studies in Group B (psychopathology allowed), two were small studies of multiple forms of abuse and one was a large study of poverty. Finally, the three studies in Group C (psychopathology allowed in participants with adversity) were of sexual abuse.

### 3.2 Meta-analysis

There was no evidence of differences in amygdala volume in any of the analyses.

**Fig. 2:**
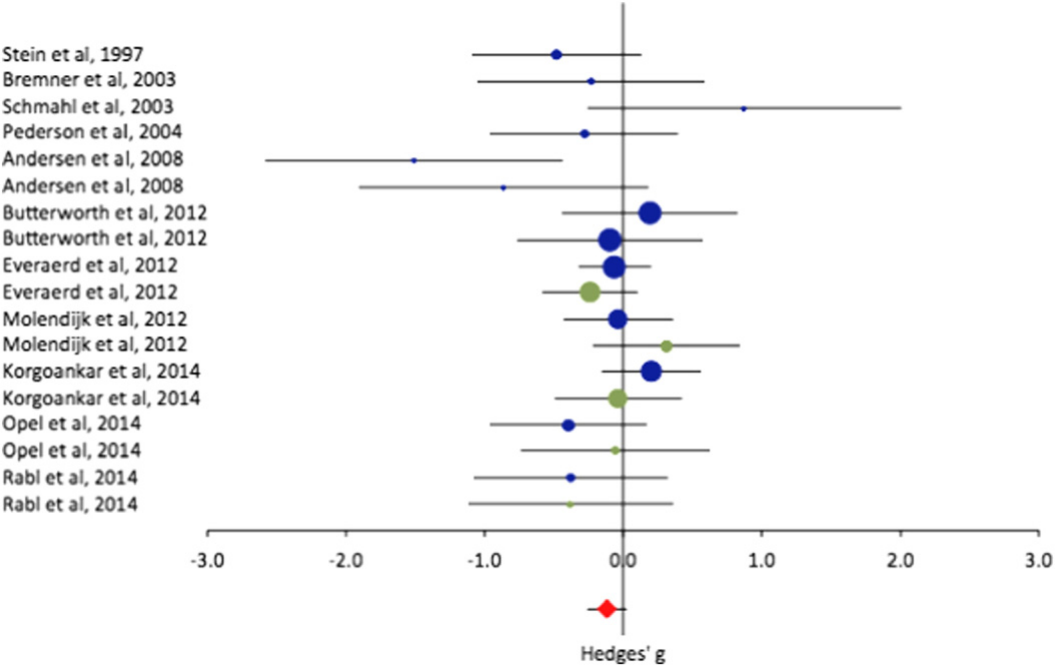
Forest plots showing effect sizes of studies of hippocampal volume differences between healthy control with and without a history of childhood adversity, controlled for gender (effect sizes from women are in blue; effect sizes from men are in green). Positive effect sizes indicate the region has increased volume in those with childhood adversity, negative effects sizes indicate the region has decreased volume in those with childhood adversity. For each study, the circle indicates the effect size, and the horizontal lines indicate the 95% confidence intervals. The size of the circle represents the relative weight of the particular study in the overall meta-analysis. The diamond at the bottom of each graph represents the overall effect calculated using a random effects model.

Compared with those without childhood adversity, people with childhood adversity had decreased volumes of the hippocampus (Fig. 1 and Table 2) (hedges g = − 0.15, *p* = 0.010). When the same analysis was carried out in the eleven studies for which data by gender were available, the evidence for an association was weaker (hedges g = − 0.17, *p* = 0.052) and, when controlled for gender, even less evident (hedges g = − 0.12, *p* = 0.124) (Fig. 2 and Table 2).

**Table 2:** Results of meta-analysis comparing controls with and without childhood adversity.

Region	Psychopathology group^f^	No. of studies	n CA +/CA −a	Comparison CA + and CA −				Heterogeneity			S.S. Bias
				Effect size^b^	95% CI	Effect size p-value^c^	% size CA + vs CA −	Q	I^2^ (%)^d^	p-value	p-value^e^
Main analysis (all studies)											
**Hippocampus**		15	783/998	− 0.15	− 0.27 to − 0.04	**0.010**	98.2	18.1	11.4	0.32	0.37
**Amygdala**		6	403/724	0.03	− 0.17 to 0.11	0.656	99.5	6.1	2.2	0.41	
Main analysis (studies with data by gender available)											
**Hippocampus**		11	505/929	− 0.17	− 0.35 to 0.00	0.052	97.9	15.9	30.9	0.14	
**Amygdala**		4	162/626	0.01	− 0.27 to 0.28	0.966	99.9	5.5	45.4	0.14	
Main analysis, controlling for gender											
**Hippocampus**		11	505/929	− 0.12	− 0.27 to 0.03	0.124	98.6	22.3	23	0.17	
**Amygdala**		5	162/626	0.08	− 0.13 to 0.29	0.459	101.3	9.1	12	0.34	
Analysis stratified by psychopathology											
**Hippocampus**	A	9	657/572	− 0.15	− 0.26 to − 0.03	**0.015**	98.6	4.0	0	0.91	0.61
	B	3	72/380	0.06	− 0.36 to 0.47	0.790	100.4	2.9	31.9	0.23	
	C	3	54/46	− 0.66	− 1.14 to − 0.18	**0.007**	93.9	3.9	22.7	0.27	0.31
**Amygdala**	A	4	348/361	− 0.09	− 0.24 to 0.06	0.240	98.6	1.2	0	0.88	
	B	2									
	C	0									
Analysis stratified by psychopathology, controlling for gender											
**Hippocampus**	A	5	410/472	− 0.06	− 0.20 to 0.07	0.368	99.5	7.9	0	0.55	
	B	3	41/411	0.05	− 0.33 to 0.43	0.800	100.6	3.3	9.4	0.35	
	C	3	54/46	− 0.66	− 1.14 to − 0.18	**0.007**	93.9	3.9	22.7	0.27	0.31
**Amygdala**	A	3	138/232	0.02	− 0.20 to 0.25	0.833	100.2	5.5	8.6	0.36	
	B	2									
	C	0									

Abbreviations: CA + childhood adversity positive. CA − childhood adversity negative; CI, confidence interval; SS, small study.

a Pooled numbers of controls with and without a history of childhood adversity.

b Hedges g, Negative effect sizes indicate that the brain structure is smaller in those with a history of childhood adversity.

c Boldface indicates significant differences in effect sizes.

d Low, 25%; moderate, 50%; and high, 75%.

e Small-study bias was calculated only when there was a significant difference.

f Studies were grouped by whether they allowed psychopathology in participants with and without childhood adversity: Group A = N/N; Group B = Y/Y, Group C = Y/N.

Studies were then analysed separately based on whether they excluded participants based on psychopathology. Within Group A (no psychopathology), childhood adversity was associated with slightly smaller volumes of the hippocampus (hedges g = − 0.15, *p* = 0.015). Within Group C (psychopathology only in those with adversity), the effect size was noticeably greater in magnitude (hedges g = − 0.66, *p* = 0.007). Within Group B (psychopathology allowed), there was no evidence of an effect (hedges g = 0.06, *p* = 0.790). These analyses by psychopathology were then run again controlling for gender. Within studies of participants with no psychopathology (Group A), there was no longer evidence of an effect (hedges g = − 0.06, *p* = 0.368). Within studies where only the adversity group had psychopathology (Group C), it was not possible to conduct this analysis as all three studies included only women (and all focussed on sexual abuse). In Group B (psychopathology allowed), there was still no evidence of an effect (hedges g = 0.05, *p* = 0.800).

We found no evidence of small study bias (which may include publication bias) in any of the analyses.

### 3.3 Sensitivity analysis

Throughout the different analyses, using percentage change as the effect size did not alter whether a result was significant or not. There was also no change in the results when the correlation coefficient between left and right regions was set to 0, 0.5 or 1.

## 4 Discussion

In this meta-analysis of general population and control samples, we found evidence for smaller hippocampal volume in those with a history of childhood adversity compared with those without. However, this effect was small (hedges g = − 0.15, *p* = 0.010) and was not evident after accounting for gender in the subset of studies for which data by gender was available. Conducting this analysis separately based on psychopathology showed evidence of a strong association between childhood adversity and smaller hippocampal volume in studies where participants with childhood adversity had psychopathology but those without did not; a smaller association in studies where neither group had psychopathology, and no evidence of an association in studies where both groups had psychopathology. However, these groups of studies differed in what type of childhood adversity they predominantly studied - sexual abuse, multiple forms of abuse and childhood poverty, respectively – and also by gender. No associations between childhood adversity and amygdala volume were found.

Several limitations should be mentioned. The number of studies included was sizeable but may not have been sufficient to capture what may be a small effect in a relatively resilient sample. Several different forms of childhood adversity were included, from poverty to sexual abuse, and there were not enough studies to allow consideration of different forms of adversity separately, which may impact differentially on brain structures. Evidence has started to accumulate for specificity of certain types of adversity (Everaerd et al., 2016). The current meta-analysis was also not able to investigate the impact of adversity at different ages within childhood. This would have been useful as evidence is accumulating for the existence of sensitive periods for the impact of childhood adversity on the hippocampus (Andersen et al., 2008, Pechtel et al., 2014). Similarly, it was not possible with the data available to restrict our analysis to more severe forms of abuse or to one or the other side of each brain structure. Finally, it is not possible to establish the direction of causality from studies of retrospectively reported childhood adversity. A recent paper suggests that childhood adversity is preceded by lower cognitive abilities (Danese et al., 2016); this could also be the case for hippocampal volume, given its key role in cognition.

The initial finding of a smaller hippocampus in those with a history of childhood adversity is in line with previous studies using other imaging analysis approaches. The present meta-analysis was focussed on region of interest studies, where the volume and standard deviation of specific brain structures could be extracted or requested from authors. As an alternative, whole brain studies using voxel-based morphometry allow for unbiased and unconstrained characterisation of the impact of childhood adversity. An important meta-analysis (Lim et al., 2014) of voxel-based morphometry studies reported an association between childhood maltreatment and smaller amygdala and parahippocampal gyrus volumes. It is possible that these results were confounded to an extent by psychopathology, as although the groups were defined on the basis of maltreatment, they also differed by psychiatric diagnosis. In the twelve studies included, only one of the maltreatment groups did not include people with psychiatric comorbid disorders, compared with nine of the non-maltreated (control) groups. The current study attempted to account for the role of psychiatric history but this task was complicated by the heterogeneity of the studies, which were quite different not only in prevalence of psychopathology but in type of adversity and gender. The largest effect size was observed in 3 studies which included patients with psychopathology within the adversity group, but no psychopathology in those with no adversity. These publications were all studies of sexual abuse in women. The studies where no psychopathology was allowed showed a weaker association. The groups where psychopathology was permitted in both groups showed no association; this group was composed of one very large study of poverty and two small studies of multiple forms of abuse. Therefore type of abuse could have been driving these differences, with sexual abuse having a particularly strong impact on hippocampal and childhood poverty having less impact. A meta-analysis (Riem et al., 2015) of hippocampal volume and childhood maltreatment irrespective of psychopathology found a strong association, possibly driven in part by the inclusion of psychiatric samples. That meta-analysis found no evidence that sexual abuse was a particularly strong driver of this association.

The lack of association between maltreatment and hippocampal volume after controlling for gender may have arisen because this second analysis included fewer studies. In fact, when the main analysis was carried out in the 11 studies for which data by gender was available, the p-value was bigger (*p* = 0.052). Alternatively, that this association was not evident in the gender-controlled analysis suggests some degree of confounding by gender. This highlights the need to consider gender as a confounder when examining the impact of childhood adversity.

Given gender differences in neurodevelopmental trajectories in childhood and adolescence (Lenroot and Giedd, 2010); (Rijpkema et al., 2012), similar childhood adversities may impact on specific brain regions differently by gender (Tottenham and Sheridan, 2009). The importance of examining this issue separately by gender is further supported by a recent study reporting sex-differences in the effect of childhood adversity on brain structure (Everaerd et al., 2016). More specifically, previous studies suggest that male hippocampi are more vulnerable to stress (Everaerd et al., 2012). Similar findings have been found for neglect (Frodl et al., 2010) and emotional abuse (Samplin et al., 2013). This greater resilience to stress in women has been suggested to be due to a protective effect of oestrogen, as demonstrated in trials of oestrogen replacement therapy (Lord et al., 2008, Sherwin and McGill, 2003). Therefore, future studies may consider stratifying their results by gender. They should also take into account differences of type of abuse by gender, with sexual abuse being more common in women (Barth et al., 2013).

The hippocampus and the amygdala are especially relevant to the study of the impact of childhood adversity because they are densely populated with glucocorticoid receptors and highly susceptible to the effects of glucocorticoids via damage, dendritic atrophy and neurogenesis suppression (Sapolsky, 2003). These brain structures are also of interest because there is now meta-analytical evidence that they are altered in various psychiatric disorders such as PTSD (O'Doherty et al., 2015), depression and bipolar disorder (Wise et al., 2016) (hippocampus only), schizophrenia (Shepherd et al., 2012) and borderline personality disorder (Ruocco et al., 2012). There is evidence that some of the abnormalities in these brain structures that have been ascribed to psychopathology may be due to increased childhood adversity in these psychiatric populations relative to healthy controls. For example, (Opel et al., 2014) reports that the smaller hippocampal volume found in people with major depressive disorder relative to controls was only present in the subgroup of depressed participants with a history of childhood adversity.

The lack of association between childhood adversity and amygdala volume may be due to the fact that there were fewer studies that included this brain structure. Some studies have suggested that amygdala volume may be increased in those who have experienced neglect but reduced in those who experience abuse (Teicher and Samson, 2016), and that therefore any association is undetected when all forms of adversity are analysed together. However, when studies were analysed separately based on psychopathology, the only group with enough studies for the amygdala analysis was of studies of participants with no psychopathology; these four studies were all of abuse and no significant association was observed.

For future meta-analysis where additional studies are available it may be possible to focus on specific sub-regions of brain structures. For example, it is now possible to focus on specific hippocampal subfields that translational research indicates are most strongly affected by stress. A general population study (Teicher et al., 2012) found associations between childhood adversity and reduced volume of two such subfields of the hippocampus: the dentate gyrus (sensitive to glucocorticoid neurogenesis suppression) and the CA3 subfield of the cornu ammonis (sensitive to glucocorticoid dendritic remodelling).

In conclusion, there is evidence of slight reduction in hippocampal volume in general population and healthy control samples, though less marked than those found in psychiatric populations. This lends support to the idea of a greater sensitivity to adversity and stress in those who go on to develop psychiatric disorders. This meta-analysis also highlights the need for studies to report and account for gender differences in history and impact of childhood adversity, as well as psychiatric comorbidities in “healthy control” samples.

The following are the supplementary data related to this article.

Supplementary material Image 1

Supplementary Table 1 Further information on studies included in the meta-analysis. Supplementary Table 1

Supplementary Fig. 1 PRISMA flowchart showing selection of articles for analysis (Moher et al., 2009). Supplementary Fig. 1
