# Supplementary material for: Meta-analysis of associations between childhood adversity and hippocampus and amygdala volume in non-clinical and general population samples
Source: Neuroimage Clin. 2017 Feb 22;14:471–9. doi: 10.1016/j.nicl.2017.02.016 (PMC5331153; doi:10.1016/j.nicl.2017.02.016)
Supplement: Supplementary file 1 — Supplementary material [file mmc1.docx]

Supplementary material – search terms

(developmental OR early life OR child*[Title/Abstract]) AND (abuse[Title/Abstract] OR trauma[Title/Abstract] OR maltreatment[Title/Abstract] OR neglect[Title/Abstract] OR adversity[Title/Abstract] OR child abuse[MeSH Terms] OR adult survivors of child abuse[MeSH Terms]) AND mri

Pubmed-generated search details:

(developmental[All Fields] OR (early[All Fields] AND ("life"[MeSH Terms] OR "life"[All Fields])) OR (child[Title/Abstract] OR child'[Title/Abstract] OR child''[Title/Abstract] OR child'head[Title/Abstract] OR child'ren[Title/Abstract] OR child's[Title/Abstract] OR child's'[Title/Abstract] OR child'shealth[Title/Abstract] OR child'stalk[Title/Abstract] OR child'svoice[Title/Abstract] OR child2015[Title/Abstract] OR childa[Title/Abstract] OR childacute[Title/Abstract] OR childadolescent[Title/Abstract] OR childage[Title/Abstract] OR childand[Title/Abstract] OR childas[Title/Abstract] OR childattention[Title/Abstract] OR childbaring[Title/Abstract] OR childbase[Title/Abstract] OR childbearer[Title/Abstract] OR childbearers[Title/Abstract] OR childbearers'[Title/Abstract] OR childbearimg[Title/Abstract] OR childbearing[Title/Abstract] OR childbearing'[Title/Abstract] OR childbearing's[Title/Abstract] OR childbearingage[Title/Abstract] OR childbed[Title/Abstract] OR childbeds[Title/Abstract] OR childbirt[Title/Abstract] OR childbirth[Title/Abstract] OR childbirth'[Title/Abstract] OR childbirth''[Title/Abstract] OR childbirth's[Title/Abstract] OR childbirthing[Title/Abstract] OR childbirthis[Title/Abstract] OR childbirthreport[Title/Abstract] OR childbirths[Title/Abstract] OR childbirthwas[Title/Abstract] OR childblains[Title/Abstract] OR childbood[Title/Abstract] OR childbraininjurytrust[Title/Abstract] OR childbred[Title/Abstract] OR childbreeding[Title/Abstract] OR childbrith[Title/Abstract] OR childcare[Title/Abstract] OR childcare'[Title/Abstract] OR childcare's[Title/Abstract] OR childcare1the[Title/Abstract] OR childcarers[Title/Abstract] OR childcarers'[Title/Abstract] OR childcaring[Title/Abstract] OR childcentred[Title/Abstract] OR childchecks[Title/Abstract] OR childchood[Title/Abstract] OR childd[Title/Abstract] OR childdagger[Title/Abstract] OR childdata[Title/Abstract] OR childdeath[Title/Abstract] OR childdecode[Title/Abstract] OR childdevelopment[Title/Abstract] OR childdhood[Title/Abstract] OR childdren[Title/Abstract] OR childdynha[Title/Abstract] OR childe[Title/Abstract] OR childed[Title/Abstract] OR childeen[Title/Abstract] OR childen[Title/Abstract] OR childen's[Title/Abstract] OR childer[Title/Abstract] OR childeren[Title/Abstract] OR childeren's[Title/Abstract] OR childern[Title/Abstract] OR childern's[Title/Abstract] OR childerns[Title/Abstract] OR childers[Title/Abstract] OR childers'[Title/Abstract] OR childes[Title/Abstract] OR childesigns[Title/Abstract] OR childevrepen[Title/Abstract] OR childfamilyconnections[Title/Abstract] OR childfeeding[Title/Abstract] OR childfile[Title/Abstract] OR childfocused[Title/Abstract] OR childfood[Title/Abstract] OR childfree[Title/Abstract] OR childfrom[Title/Abstract] OR childfs[Title/Abstract] OR childfund[Title/Abstract] OR childgood[Title/Abstract] OR childgrowth[Title/Abstract] OR childh[Title/Abstract] OR childhaven's[Title/Abstract] OR childhcod[Title/Abstract] OR childhead[Title/Abstract] OR childhealth[Title/Abstract] OR childhealthresearch[Title/Abstract] OR childhelp[Title/Abstract] OR childhelp's[Title/Abstract] OR childhhod[Title/Abstract] OR childhhood[Title/Abstract] OR childhod[Title/Abstract] OR childhodd[Title/Abstract] OR childhoiod[Title/Abstract] OR childhold[Title/Abstract] OR childhon[Title/Abstract] OR childhoo[Title/Abstract] OR childhooc[Title/Abstract] OR childhood[Title/Abstract] OR childhood'[Title/Abstract] OR childhood''[Title/Abstract] OR childhood'all[Title/Abstract] OR childhood's[Title/Abstract] OR childhood2000[Title/Abstract] OR childhood2010[Title/Abstract] OR childhoodacute[Title/Abstract] OR childhoodfrom[Title/Abstract] OR childhoodheadache[Title/Abstract] OR childhoodobesity[Title/Abstract] OR childhoodpulmonary[Title/Abstract] OR childhoods[Title/Abstract] OR childhoodtraumatic[Title/Abstract] OR childhoodwas[Title/Abstract] OR childhoodwith[Title/Abstract] OR childhoof[Title/Abstract] OR childhoofd[Title/Abstract] OR childhool[Title/Abstract] OR childhoold[Title/Abstract] OR childhoond[Title/Abstract] OR childhoood[Title/Abstract] OR childhoos[Title/Abstract] OR childhoot[Title/Abstract] OR childhope[Title/Abstract] OR childhospice[Title/Abstract] OR childhours[Title/Abstract] OR childhren[Title/Abstract] OR childi[Title/Abstract] OR childia[Title/Abstract] OR childiae[Title/Abstract] OR childiidae[Title/Abstract] OR childinfo[Title/Abstract] OR childis[Title/Abstract] OR childisch[Title/Abstract] OR childish[Title/Abstract] OR childish'[Title/Abstract] OR childishly[Title/Abstract] OR childishness[Title/Abstract] OR childism[Title/Abstract] OR childist[Title/Abstract] OR childkind[Title/Abstract] OR childlen[Title/Abstract] OR childlern[Title/Abstract] OR childless[Title/Abstract] OR childless'[Title/Abstract] OR childlessness[Title/Abstract] OR childlessness'[Title/Abstract] OR childlex[Title/Abstract] OR childlhood[Title/Abstract] OR childlike[Title/Abstract] OR childlikeness[Title/Abstract] OR childline[Title/Abstract] OR childline's[Title/Abstract] OR childliver[Title/Abstract] OR childliverdisease[Title/Abstract] OR childloss[Title/Abstract] OR childm[Title/Abstract] OR childmeds[Title/Abstract] OR childminder[Title/Abstract] OR childminder's[Title/Abstract] OR childminders[Title/Abstract] OR childminders'[Title/Abstract] OR childminding[Title/Abstract] OR childmother[Title/Abstract] OR childneph[Title/Abstract] OR childness[Title/Abstract] OR childnessness[Title/Abstract] OR childnood[Title/Abstract] OR childobesity180[Title/Abstract] OR childomics[Title/Abstract] OR childonium[Title/Abstract] OR childood[Title/Abstract] OR childoriented[Title/Abstract] OR childover[Title/Abstract] OR childparent[Title/Abstract] OR childpedsql[Title/Abstract] OR childpersonality[Title/Abstract] OR childplay[Title/Abstract] OR childprev[Title/Abstract] OR childproof[Title/Abstract] OR childproofed[Title/Abstract] OR childproofing[Title/Abstract] OR childproofing'[Title/Abstract] OR childprotective[Title/Abstract] OR childpsychiatric[Title/Abstract] OR childpsychiatrist[Title/Abstract] OR childpsychiatrists[Title/Abstract] OR childpsychiatry[Title/Abstract] OR childpsychotherapeutic[Title/Abstract] OR childptsd[Title/Abstract] OR childpugh[Title/Abstract] OR childr[Title/Abstract] OR childraising[Title/Abstract] OR childre[Title/Abstract] OR childreach[Title/Abstract] OR childrean[Title/Abstract] OR childrearers[Title/Abstract] OR childrearing[Title/Abstract] OR childrearing'[Title/Abstract] OR childred[Title/Abstract] OR childredn[Title/Abstract] OR childreen[Title/Abstract] OR childrelationships[Title/Abstract] OR childrem[Title/Abstract] OR children[Title/Abstract] OR children'[Title/Abstract] OR children''[Title/Abstract] OR children''s[Title/Abstract] OR children'car[Title/Abstract] OR children'emotional[Title/Abstract] OR children'rights[Title/Abstract] OR children's[Title/Abstract] OR children's'[Title/Abstract] OR children's'hands[Title/Abstract] OR children'scatalgine[Title/Abstract] OR children'shealth[Title/Abstract] OR children'significantly[Title/Abstract] OR children'spsychiatricservices[Title/Abstract] OR children3[Title/Abstract] OR children7[Title/Abstract] OR childrena[Title/Abstract] OR childrenae[Title/Abstract] OR childrenaged[Title/Abstract] OR childrenand[Title/Abstract] OR childrenas[Title/Abstract] OR childrenat[Title/Abstract] OR childrencompared[Title/Abstract] OR childrendagger[Title/Abstract] OR childrendata[Title/Abstract] OR childrendiagnosis[Title/Abstract] OR childrendisease[Title/Abstract] OR childrenduring[Title/Abstract] OR childrenese[Title/Abstract] OR childrenfirst[Title/Abstract] OR childrenfrom[Title/Abstract] OR childrenfs[Title/Abstract] OR childrenhad[Title/Abstract] OR childrenhood[Title/Abstract] OR childreni[Title/Abstract] OR childrenii[Title/Abstract] OR childrenin[Title/Abstract] OR childreninterview[Title/Abstract] OR childrenirmina[Title/Abstract] OR childrenis[Title/Abstract] OR childrenmore[Title/Abstract] OR childrenn[Title/Abstract] OR childrenof[Title/Abstract] OR childrenos[Title/Abstract] OR childrenover[Title/Abstract] OR childrenpen[Title/Abstract] OR childrenpsychologysubjectivityteenage[Title/Abstract] OR childrenrsquo[Title/Abstract] OR childrens[Title/Abstract] OR childrens'[Title/Abstract] OR childrens's[Title/Abstract] OR childrenshospital[Title/Abstract] OR childrensi[Title/Abstract] OR childrensmemorial[Title/Abstract] OR childrensnational[Title/Abstract] OR childrensoncologygroup[Title/Abstract] OR childrenspainguideline[Title/Abstract] OR childrensuspected[Title/Abstract] OR childrensustaining[Title/Abstract] OR childrenthis[Title/Abstract] OR childrentm[Title/Abstract] OR childrentrade[Title/Abstract] OR childrenwas[Title/Abstract] OR childrenwere[Title/Abstract] OR childrenwhen[Title/Abstract] OR childrenwho[Title/Abstract] OR childrenwith[Title/Abstract] OR childrenwithin[Title/Abstract] OR childrenxmedian[Title/Abstract] OR childrepresents[Title/Abstract] OR childreq[Title/Abstract] OR childrerl[Title/Abstract] OR childrern[Title/Abstract] OR childres[Title/Abstract] OR childresn's[Title/Abstract] OR childress[Title/Abstract] OR childress'[Title/Abstract] OR childress's[Title/Abstract] OR childressi[Title/Abstract] OR childressness[Title/Abstract] OR childrestrains[Title/Abstract] OR childreti[Title/Abstract] OR childrey[Title/Abstract] OR childrhood[Title/Abstract] OR childrive[Title/Abstract] OR childrn[Title/Abstract] OR childrn's[Title/Abstract] OR childrne[Title/Abstract] OR childroom[Title/Abstract] OR childrren[Title/Abstract] OR childs[Title/Abstract] OR childs'[Title/Abstract] OR childs'b[Title/Abstract] OR childsafe[Title/Abstract] OR childsaving[Title/Abstract] OR childscore[Title/Abstract] OR childself[Title/Abstract] OR childseq[Title/Abstract] OR childserv[Title/Abstract] OR childsight[Title/Abstract] OR childsii[Title/Abstract] OR childsish[Title/Abstract] OR childsitters[Title/Abstract] OR childsmile[Title/Abstract] OR childsmile'[Title/Abstract] OR childsmile's[Title/Abstract] OR childspacing[Title/Abstract] OR childspla[Title/Abstract] OR childsplay[Title/Abstract] OR childsubtotal[Title/Abstract] OR childtending[Title/Abstract] OR childtrade[Title/Abstract] OR childwards[Title/Abstract] OR childwatch[Title/Abstract] OR childwelfare[Title/Abstract] OR childwish[Title/Abstract] OR childwood[Title/Abstract] OR childx[Title/Abstract])) AND (abuse[Title/Abstract] OR trauma[Title/Abstract] OR maltreatment[Title/Abstract] OR neglect[Title/Abstract] OR adversity[Title/Abstract] OR "child abuse"[MeSH Terms] OR "adult survivors of child abuse"[MeSH Terms]) AND ("magnetic resonance imaging"[MeSH Terms] OR ("magnetic"[All Fields] AND "resonance"[All Fields] AND "imaging"[All Fields]) OR "magnetic resonance imaging"[All Fields] OR "mri"[All Fields])
