# Supplementary material for: Meta-analysis of associations between childhood adversity and hippocampus and amygdala volume in non-clinical and general population samples
Source: Neuroimage Clin. 2017 Feb 22;14:471–9. doi: 10.1016/j.nicl.2017.02.016 (PMC5331153; doi:10.1016/j.nicl.2017.02.016)
Supplement: Supplementary Table 1 — Further information on studies included in the meta-analysis. [file mmc2.docx]

Supplementary Table 1

|  | Study | Mean (SD) age at interview | n (%) female | | n (%) with a psychiatric illness | | MRI field strength |
| --- | --- | --- | --- | --- | --- | --- | --- |
|  |  |  | CA+ | CA+ | CA+ | CA- |  |
| 1 | Stein et al, 1997 | CA+ 32.0 (6.3), CA- 30.2 (6.4) | 21 (100) | 21 (100) | 15 (71%) current PTSD, 15 (71%) dissociative disorder (1 dissociative amnesia, 5 dissociative identity disorder, 9 dissociative disorder NOS). 6 (28%) current MDD, 1 social phobia, 1 OCD | No current Axis I pathology. | 1.5 |
| 2 | Bremner et al., 2003 | CA+ 32 (8), CA- 38 (7) | 12 (100) | 11 (100) | 3 (25%) lifetime MDD, 0 current MDD, 1 (8%) each current panic disorder with agoraphobia, lifetime OCD, GAD, lifetime anorexia, lifetime marijuana dependence, lifetime marijuana abuse, lifetime cocaine dependence | No psychiatric history | 1.5 |
| 3 | Schmahl et al, 2003 | 31.5 (8.0) | 5 (100) | 10 (100) | NR | NR | 1.5 |
| 4 | Pederson et al., 2004 | CA+ 26.8 (6.6), CA- 23.8 (5.6) | 17 (100) | 17 (100) | 50% major depressive symptoms | 12.3% major depressive symptoms | 1.5 |
| 5 | Cohen et al, 2006 | 39.9 (17.2) | NR | NR | NR | NR | 1.5 |
| 6 | Andersen et al., 2008 | 19.7 (1.4) | 31 (100) | 14 (100) | No current or past: 7 (26.9%). Current: PTSD 4 (15.4%), MDD 3 (11.5%), one each (7.7%) of DD, SP, GAD, OCD, BE, ADHD. Past: MDD 14 (53.8%), PTSD 4 (15.4%), DD 2 (7.7%), OCD 2 (7.7%), CU 2 (7.7%), one each (3.8%) of ADHD, BD, SAD, BN. | 0% | 1.5 |
| 7 | Frodl et al., 2010 | 41.9 (13.2) | NR | NR | None | None | 1.5 |
| 8 | Bermingham et al., 2012 | By BICCI group: T-carriers (n=18) 36.6 (11.8); CC (n=26) 35.3 (13.4) | NR | NR | NR | NR | 3 |
| 9 | Butterworth et al, 2012 | 46.7 (1.41) | 10 (52.6) | 215 (56.0) | NR | NR | 1.5 |
| 10 | Everaerd et al., 2012 | males 24.3 (6.3), females 23.3 (5.1) | 113 (58.9) | 108 (65.5) | NR | NR | 3 |
| 11 | Molendijk et al., 2012 | NR | 50 (62.5) | 49 (65.3) | None | None | 3 |
| 12 | Baker et al., 2013 | 34.72 (17.0) | 32 (41.8) | 50 (51.4) | None | None | 1.5 |
| 13 | Korgaonkar et al., 2013 | NR | 50 (67.6) | 76 (50.7) | None | None | 1.5 |
| 14 | Opel et al., 2014 | 37.2 (11.8) | 20 (58.8) | 31 (60.8) | None | None | 3 |
| 15 | Rabl et al., 2014 | 23.79 (3.03) | 17 (56.7) | 15 (48.4) | None | None | 3 |

**Supplementary Table 1**. Further information on studies included in the meta-analysis.

Abbreviations: NR not reported. CA+ childhood adversity positive. CA- childhood adversity negative. hc hippocampus. am amygdala. PTSD post-traumatic stress disorder. NOS not otherwise specified. MDD major depressive disorder. OCD obsessive-compulsive disorder. GAD generalised anxiety disorder. DD depersonalisation disorder. SP social phobia. BE binge eating. ADHD attention deficit/hyperactivity disorder. CU cannabis use. BD bipolar disorder. SAD seasonal affective disorder. BN bulimia nervosa.
