# Supplementary material for: Meta-analysis of associations between childhood adversity and hippocampus and amygdala volume in non-clinical and general population samples
Source: Neuroimage Clin. 2017 Feb 22;14:471–9. doi: 10.1016/j.nicl.2017.02.016 (PMC5331153; doi:10.1016/j.nicl.2017.02.016)
Supplement: Supplementary Fig. 1 — PRISMA flowchart showing selection of articles for analysis (Moher et al., 2009). [file mmc3.doc]

**Screening**

**Included**

**Eligibility**

**Identification**

Records identified through database searching
(n = 1458)

Additional records identified through other sources
(n = 3)

Records after duplicates removed
(n = 1461)

Records screened
(n = 1461)

Records excluded
(n = 1344)

Not about childhood adversity: 1058

No volumetric data: 211

Review: 75

Full-text articles assessed for eligibility
(n = 117)

Full-text articles excluded
(n = 102)

No suitable control group: 36

No volumetric data: 26

No hippocampus/amygdala volumes: 17

Sample used in another study: 12

Adolescents or children only: 4

Elderly people only: 1

War veterans only: 3

No response from author: 3

Studies included
(n = 15)

Supplementary Figure 1: PRISMA flowchart showing selection of articles for analysis (Moher, Liberati, Tetzlaff, Altman, & Grp, 2009).
